# Sensory motor mechanisms unify psychology: the embodiment of culture

**DOI:** 10.3389/fpsyg.2013.00885

**Published:** 2013-11-29

**Authors:** Tamer Soliman, Alison Gibson, Arthur M. Glenberg

**Affiliations:** Department of Psychology, Arizona State UniversityTempe, AZ, USA

**Keywords:** embodied cognition, distance perception, motor effort, culture, self-construal

## Abstract

Sensorimotor mechanisms can unify explanations at cognitive, social, and cultural levels. As an example, we review how anticipated motor effort is used by individuals and groups to judge distance: the greater the anticipated effort the greater the perceived distance. Anticipated motor effort can also be used to understand cultural differences. People with interdependent self- construals interact almost exclusively with in-group members, and hence there is little opportunity to tune their sensorimotor systems for interaction with out-group members. The result is that interactions with out-group members are expected to be difficult and out-group members are perceived as literally more distant. In two experiments we show (a) interdependent Americans, compared to independent Americans, see American confederates (in-group) as closer; (b) interdependent Arabs, compared to independent Arabs, perceive Arab confederates (in- group) as closer, whereas interdependent Americans perceive Arab confederates (out-group) as farther. These results demonstrate how the same embodied mechanism can seamlessly contribute to explanations at the cognitive, social, and cultural levels.

## Toward a unified psychology

Academic psychology compartmentalized the mind into cognitive, social and cultural partitions, and developed for each a self-delimited conceptual paradigm and explanatory tradition. Typically, the cognitive, social, and cultural psychologists believe that they target three different mental structures in the minds of the same people. For the first, the research participants are computer-like information processors (e.g., Newell, 1980), for the second, they are social agents driven by basic motivations to fulfill interpersonal goals (Forgas et al., 2005), and, for the third, they are normative populations immersed each in their local system of values, beliefs, and worldviews (e.g., Shweder, 1996). With these disparate levels of construct specification, cross-talk over the epistemological fence is limited (e.g., Messick and Mackie, 1989; Hong et al., 2000; Nisbett, 2003; Knoblich and Sebanz, 2006). In these accounts, the levels of the mind may, at best, interact, but remain conceptually intact, much like billiard-balls that maintain their self-contained identities through their collisions.

Our goal is to take steps toward a unified account of the human mind by finding theoretical units of analysis that apply equally to understanding the cognitive, social, and cultural aspects of behavior. Alongside others (e.g., Schubert and Semin, 2009; Glenberg, 2010), we believe that the body has this unification potential; its sensorimotor mechanisms can explain behavior that plays out in a physical, social, or cultural context. Our strategy is to use the bodily level of description to side-step the three different characterizations of the mind found in the three sub-disciplines, and thereby demonstrate the possibility of specifying level-neutral mechanisms that could uniformly explain cognitive, social, and cultural behavior.

Specifically, the empirical plan is to identify a sensorimotor mechanism with proven explanatory power at one of these levels, then to examine whether this same mechanism can predict behavioral patterns that are well established at the other two levels. Fortunately, one such mechanism has already been characterized and its cognitive (Proffitt, 2006) and social (Schnall et al., 2008) effects successfully demonstrated. After reviewing these, we present our own account to explain how the mechanism can generate plausible predictions in a cultural context, then we report on two studies that generally confirmed our predictions.

### Motor effort in basic cognitive and social processes

In cognitive psychology, Proffitt and his associates forged a link between two characteristics of the motor system, and they used this link to propose a novel reformulation of the mechanism of distance perception (Proffitt, 2006). On Proffitt's account, the voluntary muscle system is sensitive to the bioenergetic status of the body (Davis et al., 1997; Achten et al., 2004; Coyle, 2004) while being simultaneously tightly coupled with the visual system (Hommel et al., 2001). On the basis of this link, it was proposed that distance perception could not only be conceptualized as an algorithmic process determined exclusively by visual cues (e.g., Cutting and Vishton, 1995), but that it is an ecological integrative process in which the motor system plays an important role. Specifically, Proffitt predicts that visual perception of distance to a target should be scaled by the motor effort required to interact with (e.g., walk up to) that target.

The hypothesized effect of motor effort was confirmed (see Proffitt, 2006, for a review): participants reported inflated visual distance to targets that required more motor effort to reach. Participants who were wearing a backpack, exhausted, in poor fitness, elderly, or in ill-health reported hills to appear steeper when compared with their fit, healthy, younger, or rested counterparts.

Could the same sensorimotor mechanism extend to the realm of the social? Schnall and her colleagues (Schnall et al., 2008) argued that a supportive other is construed by the body as a potential resource, either providing a surplus of energy or easing the burden on internal resources. Thus, social support enhances the efficacy of the individual's motor and cognitive systems during task performance. To test this hypothesis, a group of solo participants and another group accompanied by their friends were asked to estimate the slope of hills. Others were asked to imagine the presence of a friend, a neutral individual, or a disliked person before offering their estimates. In both experiments, the real or imagined presence of a (potentially) supportive other led to smaller estimates of the hill's slope.

### Motor effort and cultural orientations of independence and interdependence

To push the explanatory and predictive power of this mechanism into the domain of cultural behavior, we brought together findings from several lines of research. The first is that the motor system is involved in interpersonal interactions. Of course the motor system is needed to talk, to observe (e.g., move the eyes), to move toward, and to cooperate in physical tasks. But additionally, the motor system is used to help recognize the goals of others using an automatic resonance process based on their movements (Rizzolatti et al., 2001; Blakemore and Frith, 2005; Wilson and Knoblich, 2005). When person A observes person B act, A's motor repertoire (predominately in premotor cortex) is automatically activated, or resonates, and provides a model of what B is doing. When successful, this resonance generates A's goals when engaging in this action, and A uses these goals as an understanding of B's goals. Note that this simulation, or resonance, is not in anticipation of the B's actions, but close to simultaneous with those actions.

Conversely, when the relevant motor program does not exist in A's repertoire, or when it cannot be fluently implemented, then A's recognition of the B's behavior is not as fluent, perhaps because of the more energetic investment the motor system requires to simulate the perceived action (Calvo-Merino et al., 2005; Casile and Giese, 2006; Petroni et al., 2010). For example, perception of familiar actions that the participant can fluently reproduce is accompanied by a reduced BOLD signal at the motor cortex, which is an index of low energy demands for simulating the action (Tanaka et al., 2001; Muhlau et al., 2005).

Differences in communication style across cultures are one important source of this familiarity effect on automatic motor resonance as demonstrated using fMRI to investigate modulatory effects of the perceived cultural membership on the activity of the putative human homolog of the mirror neuron system (MNS). Liew et al. (2011), for example, documented a higher BOLD signal at the MNS sites of mainland Chinese participants when watching American communicative hand gestures that were unfamiliar (e.g., “quail”) vs. familiar expressive American gestures (e.g., thumbs up). Caucasian Americans watching a Nicaraguan actor modeling either native Nicaraguan or American communicative hand gestures showed signs of more effortful motor resonance for the former (whereas the effect disappeared when the model was American; Molnar-Szakacs et al., 2007). White and black female (but not male) participants showed signs of more effortful motor resonance with the same simple finger movements when modeled by the other race compared to the same race (Desy and Theoret, 2007).

Thus, we propose that interactions with cultural out-group members are expected to be more effortful than interactions with in-group members. Note that out-group members potentially differ from in-group members along many communicative dimensions (Archer, 1997). For example, Russians may point with the middle finger, not the index finger. Also, although facial expression of emotions is qualitatively universal, differences in the rules of display (e.g., of intensity) may be misleading in cross-cultural encounters (Ekman et al., 1987; Matsumoto et al., 2002). Relative to Westerners, for example, the Japanese tend to mask both negative (Ekman, 1972) and positive (Matsumoto and Kuppersbusch, 2001) emotional expressions. Consequently, they rely (more so than Westerners) on vocally conveyed emotional tone when inferring underlying emotional states (Tanaka et al., 2010). And of course, accented pronunciation by those speaking a second language often differs significantly from native pronunciation in both segmental (place and method of articulation, e.g., Gatbonton, 1975) and supra-segmental (stress, rhythm, and intonation, e.g., Fokes and Bond, 1984) characteristics, and is actually perceived as less intelligible by native speakers (Flege, 1988). All these differences are taxing for the motor system as it attempts to resonate with observed actions [including resonance with articulatory actions, as demonstrated by Fadiga et al. (2002)].

Importantly, the more costly effort of cross-cultural encounters relative to within-cultural encounters is not only experienced during interaction, but also shapes the default expectation of interaction with out- vs. in-group members. Consistent with this assumption, a meta-analysis of the social-projection literature shows that projecting one's own state is stronger onto in-group than onto out-group others, specifically due to the perception of higher self-other similarity with the former group (Robbins and Krueger, 2005). That is, even when the interaction has not yet started, people have an expectation of less (sensorimotor, communicative) similarity with an out-group member than with an in-group member (which we will use later to justify the design of our experiments).

In addition to the proposed main effect of group-membership on the expected effort of interaction, a moderating effect needs to be added. Cross-cultural psychology suggests that people may develop an interdependent cultural orientation that stresses relatedness and harmony with their in-groups, or an independent one that emphasizes the uniqueness of their individual selves (Markus and Kitayama, 1991). People with interdependent self-construals tend to live in societies with fairly homogenous ethnic composition (e.g., East Asia), and exhibit lower levels of mobility within these settings, whereas independents typically live in ethnically diverse populations (e.g., North America) and are much more mobile relative to their interdependent counterparts (Triandis et al., 1988; Oishi and Kisling, 2009; Oishi, 2010; Schug et al., 2010). It is important to note, however, that cross-cultural psychology is moving away from identifying these categorical and geographical cultural differences in social orientation (e.g., all East Asians or all North Americans) to acknowledging that both interdependent and independent self-construals can be found, to a greater or lesser extent, around the world.

Thus, we come to the major hypothesis that drives our empirical work, namely the cultural motor-effort hypothesis. First, we suppose that cultures, and the self-construals they engender, should be conceived more as a continuum than as categories. Thus, what we describe next for interdependent and independent self-construals should be considered the ends of the continuum. Second, people who live in a predominately collectivist culture (and develop interdependent self-construals) tend to interact with family, friends, and an in-group consisting of ethnically and culturally similar people. Consequently, the motor system is strongly tuned to resonate to the behaviors of the in-group, and interaction with the in-group is smooth and relatively effortless. However, for two reasons, these interdependents are at a disadvantage when it comes to interacting with members of the out-group. Because they have little experience with out-group members, they have had little opportunity to tune their motor systems to the behaviors of out-group members. Also, because of the strong tuning or specialization for the in-group, their motor systems will have even more difficulty resonating to the different accents, gestures, etc. of the out-group than a non-tuned system. [We see this as analogous to the development of speech perception. Before an infant is strongly tuned to its native language, it can perceive phonetic distinctions that are not incorporated into the native language (e.g., Kuhl et al., 1992; Aslin et al., 1998). However, once the infant has had considerable experience with the native language, the ability to perceive non-native distinctions is lost]. Thus, interdependents experience a costly demand for motor control and prediction during cross-cultural episodes of interaction.

Third, people who live in a predominately individualistic society are forced to interact with a diversity of others. Although not as strongly tuned as interdependents to interactions with the in-group, interactions with out-group members allow these people to develop moderate skill to process and respond to people with different accents, different communicative gestures and postures, and so on. Thus, in contrast with interdependents, for independents interactions with out-group members are literally less effortful.

This hypothesis predicts that (a) interdependents anticipate motor effort upon the prospect of interacting with out-group members. This, in turn, modulates their subjective visual experience of the distance to out-group members such that their estimates of distance are inflated relative to estimates of distance to in-group members. (b) People with independent orientations should show a smaller difference in estimated distance to in-group and out-group members; they anticipate much less differential effort to interact with out-group individuals owing to the diversity of their motor social repertoire acquired by immersion in ethnically diverse settings.

Experiment 1 provides an initial, cost-effective test of the cultural motor-effort hypothesis, albeit without sampling multiple cultures. The hypothesis suggests that within any culture, those who are more interdependent will resonate more strongly with in-group members relative to those who are more independent. Thus, we predict that relative to independents, interdependents will see in-group members as closer.

The complex literature relating self-construal to prejudice (cited by a reviewer of a previous version of this article) suggests a different prediction. Some research suggests that individualism increases prejudice (e.g., Biernat et al., 1996; Katz and Hass, 1988; Sears and Henry, 2005), and a few studies (e.g., Kleugel, 1990) suggest that within a collectivist culture there is a tendency toward lower prejudice and higher tolerance toward the out-group. If prejudice can be related to motor effort, then one might expect that interdependents (from collectivist cultures) would see out-group members as closer. However, our results suggest the opposite, and so we frame those results in terms of the cultural motor-effort hypothesis.

## Study 1: distance to Americans as perceived by Americans

### Design and procedure

American participants (*n* = 33) were first trained on estimating distances to a human target in terms of seconds needed to walk to the target. Besides inducing a motor-oriented perception of distance, using seconds also minimized any potential effect of the culture-specific distance measurement units (e.g., feet vs. meters) on the reporting of perceived distance to the target, a tack that was especially important in the second study, and used here to maintain the use of a uniform DV across the experiments. The training comprised three trials. In each, the participant estimated the time to walk to the experimenter, then actually walked up to her, and finally received feedback on accuracy of the initial estimate. The training distances in this stage were quasi-randomly selected by the experimenter.

Immediately after training, but in a different location, the participant made 36 distance estimates (three 12-trial blocks) to two Caucasian (i.e., in-group) confederates^1^. The confederates stood at marks along two (imaginary) axes that intersected where the participant stood to make the estimates. The marks on each of the axes were pre-set to be at six different distances from the intersection: the short-distance marks were at 6.77 and 8.77 m from the participant's location at the intersection, the medium distances were at 10.43 and 12.43 m, and the long distances were at 20.43 and 22.43 m. The use of two distances for each of the distance ranges was meant to discourage participants from copying earlier estimates in later trials.

On any given trial, the experimenter asked the participant to turn away from both axes, one of the two confederates would position herself at a mark, then the experimenter signaled to the participant to face the confederate. The participant was then immediately asked to estimate, in seconds, the time it would take her to walk to the confederate (half of the trials), or given a 2.5 foot-long stick and asked to estimate the number of sticks it would take her to touch the confederate. On the next trial, the same process repeated, except that the other confederate would position herself on another mark on the other axis. The assignment of the two confederates to the two axes, and the order of distance presentation (i.e., short, medium, or long) were independently counterbalanced within blocks and across participants. Finally, after completing their distance estimates, the participants filled out the Interdependence and Independence subscales of the Self-Construal Survey (SCS) (Singelis, 1994).

### Analysis and results

As expected, for these American participants the mean score on the independence subscale (*M* = 5.12, *SD* = 0.73) was greater than the mean score on the interdependence subscale (*M* = 4.60, *SD* = 0.84), *t*_(28)_ = 2.51, *p* = 0.02. We used multi-level modeling (MLM) with maximum likelihood estimates of the parameters to take advantage of (a) the continuous nature of the six distances and the measure of cultural orientation, and (b) to obviate potential problems with the sphericity assumption. MLM is similar to regression in that it estimates regression parameters, however, maximum likelihood is used as the estimation procedure and estimated along with each parameter is its own standard error. Thus, the test for statistical significance is a simple *t*-test of the parameter divided by the standard error, although the degrees of freedom are often fractional because of the use of Welch-Satterthwaite estimates.

Separate MLMs were run for the two estimates of distance, namely number of seconds to walk and number of sticks. Four participants were dropped from the analysis of number of sticks, one for providing stick estimates more than 3 *SD* below the mean and three for providing stick estimates more than 3 *SD* above the mean.

The participants' cultural orientation scores were computed as the ratio of their responses to the interdependent and independent subscales of the SCS (Int:Ind). As is recommended for regression analyses that involve interaction terms (Aiken and West, 1991), all of the independent variables were centered around their respective means.

**Figure 1:**
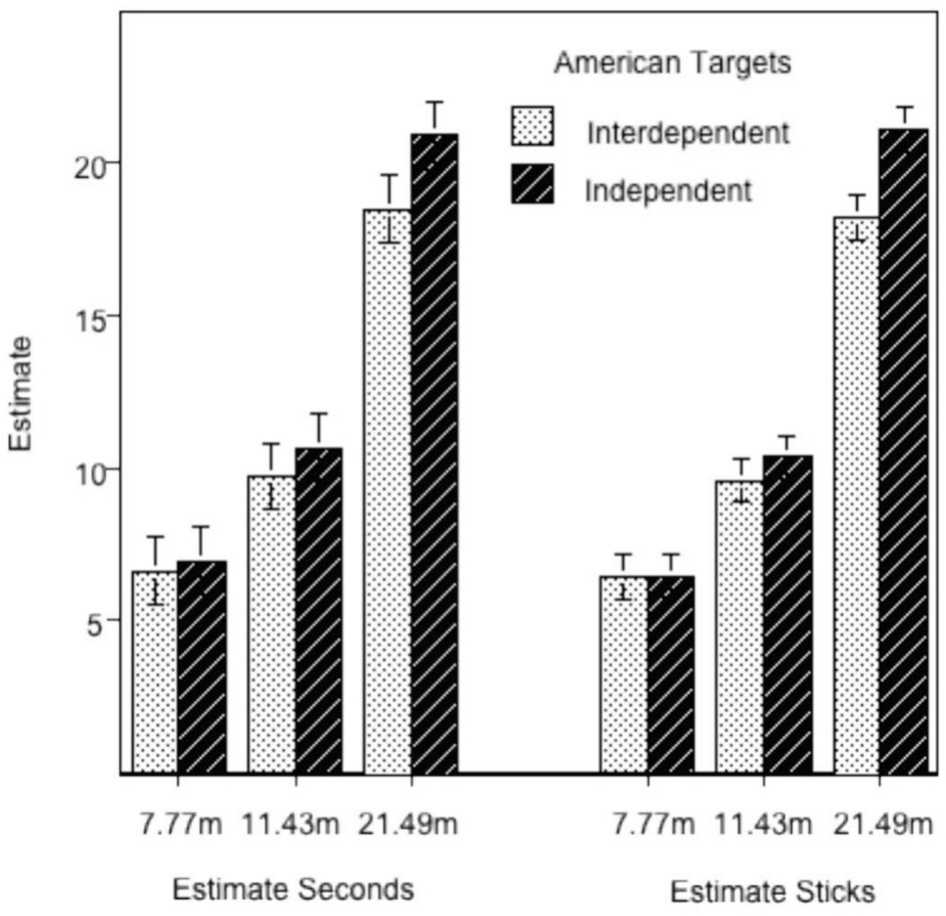
**Regression-estimated mean distance judgments to American-looking targets**. Actual distance is indicated on the abscissa. **Left:** data from Americans estimating distance as time to walk to the target; **Right:** distance estimated as number of hand-held sticks to the target.

Table 1 contains the important results from the MLMs, and Figure 1 plots the regression- estimated marginal means for the Time estimates in seconds (on the left) and Sticks (on the right) as a function of the actual distance. For both dependent variables, the effect of Distance was significant. (For Time, the parameter value of 0.858 indicates that the estimate grew by 0.858 s for each one meter increase in actual distance; likewise for Sticks, the parameter of 0.874 indicates an increase of 0.874 sticks for each meter of distance).

**Table 1:** **Parameter estimates (in seconds, upper panel) or stick-number estimates (lower panel) to walk to or touch American confederates**.

**Factor**	**Parameter**	**Seconds**		
		***df***	***t***	***p***
Int:ind	−3.000	29	−0.77	0.45
Distance	0.858	493	37.07	0.001
Int:ind × distance	−0.384	493	−3.00	0.003
		**Sticks**		
Int:ind	−3.09	29	−1.23	0.23
Distance	0.874	493.02	40.9	0.001
Int:ind × distance	−0.47	493.02	−4.41	0.001

More importantly, our predictions were confirmed in the form of significant interactions of cultural orientation (Int:Ind) and Distance for both the Time estimate and the Sticks estimate. Rather than arbitrarily breaking the sample into those with interdependent and independent self-construals and loosing the statistical power inherent in the continuous variable, we used the regression parameters to estimate means for interdependents and independents. The estimates for interdependents were obtained by using a value for the Int:Ind ratio 1 *SD* above the mean Int:Ind ratio (Aiken and West, 1991). Likewise, the values for independents were obtained by using a value of the Int:Ind ratio 1 *SD* below the mean of the Int:Ind ratio.

Turning to Figure 1, the statistical interaction becomes evident: interdependents, compared to independents, judge distance to in-group confederates as smaller. Furthermore, the difference between interdependents and independents grows with actual distance. This finding is consistent with our cultural-effort hypothesis. Namely, interdependents, compared to independents, spend more time interacting with their in-group and tuning their motor system toward those interactions. Then, because expected motor effort contributes to distance estimation (Proffitt, 2006), interdependents judge distance as smaller than independents.

The statistical interaction (that the difference in judged distance between interdependents and independents increased with distance) is even more important than the main effect for demonstrating that the groups were using different measurement scales (Proffitt and Linkenauger, 2013). That is, when the unit of measurement used by one group (e.g., X amount of anticipated effort) is different from the unit used by the other group (e.g., 3X amount of anticipated effort), then the difference in the groups' estimates becomes larger with increased distance (an interaction). For example, suppose that Person A measures distance in feet, and Person B measures distance in yards. At a distance of one yard, the two measurements, 3 (feet) and 1 (yard), differ by 2. But at a distance of 5 yards, the two measures, 15 (feet) and 5 (yards), differ by 10. Thus, the interaction is strong evidence that the interdependents and independents are measuring distance using different scales, namely different amounts of expected effort. Nonetheless, it is important to demonstrate that this interaction is replicable, and that is one purpose of the next study.

## Study 2: distance to Arabs as perceived by Arabs and americans

### Design and procedures

Clearly, our novel findings in Study 1 need to be replicated and the cultural-effort hypothesis subjected to further test. In Study 2, we used Arab-looking confederates as targets: the confederates were chosen to have dark skin tone, and one of them wore a headscarf, or hijab. Furthermore, we sampled both Arab (*n* = 16)^2^ and American (*n* = 42) participants. All other aspects of the design and procedures were identical to those of the first study, except that the participants were asked to report their estimates only in terms of time (i.e., number of seconds) to walk up to the confederates.

We predicted that the effect of cultural orientation (Int:Ind) on the American participants' estimates would flip in direction relative to the effect in Study 1. That is, since the confederates were Arab-looking, and hence, out-group members, the interdependent Americans would overestimate the distance relative to the independent Americans. Because the interdependents have tuned their motor systems to interact with other Americans, they should expect greater effort in interacting with the Arab-looking confederates than the independent Americans who have a more broadly tuned motor system. In contrast, we predicted that the Arab participants' estimates to their in-group looking confederates would resemble that of the American participants in Study 1. Interdependent Arabs have a motor system finely tuned for interaction with their in-groups, and thus they should report smaller distance to the targets than the more broadly tuned independent Arabs.

### Analysis and results

The data from three Arab participants were dropped for procedural errors, and the data from one American were dropped for providing an Int:Ind ratio more than 3 *SD* above the mean. As expected, the mean Int:Ind ratio was significantly higher for Arabs (*M* = 1.1, *SD* = 0.17) than for Americans (*M* = 0.98, *SD* = 0.14), *t*_(52)_= 2.61, *p* = 0.012.

An MLM analysis was run to examine the main effects and interactions of Distance, Int:Ind, and National culture (Arab, American). The results are reported in Table 2.

**Table 2:** **Parameter estimates (in seconds) to walk to Arab-looking confederates**.

**Factor**	**Parameter**	***df***	***t***	***p***
Int:ind	0.688	53.99	0.19	0.74
Culture	−0.43	53.99	−0.33	0.85
Distance	0.813	1884.99	74.18	0.001
Culture × int:ind	18.6	53.98	2.42	0.02
Culture × distance	−0.068	1884.99	−2.55	0.01
Int:ind × distance	−0.018	1884.99	−0.25	0.80
Culture × int:ind × distance	1.236	1884.99	7.87	0.001

**Figure 2:**
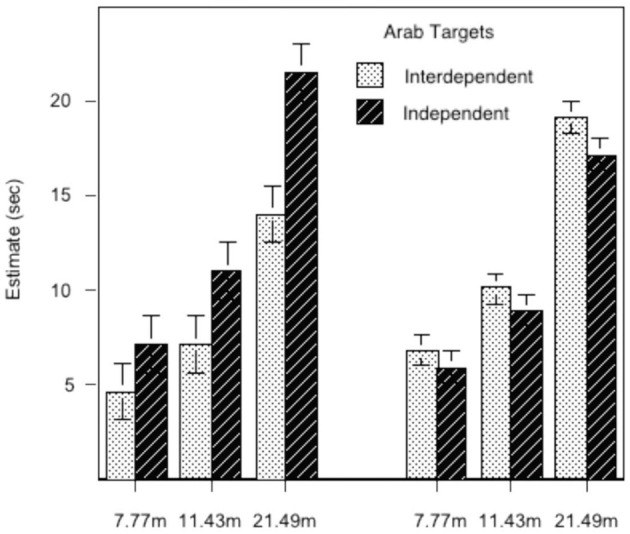
**Regression-estimated mean distance judgments (in estimated time to walk to target) to Arab-looking targets**. Actual distance is indicated on the abscissa. **Left:** data from Arabs judging distance; **Right:** data from Americans judging distance.

Figure 2 plots the regression-estimated marginal means of the participants' walking-time estimates as a function of the real distance. The predicted pattern of results was successfully obtained in the form of two interactions. First, there was an interaction of Culture (Arab vs. American) and Int:Ind on distance estimates. For the Arabs (bars on the left), the in-group (i.e., Arab-looking) confederates were perceived as closer by the interdependent than by the independent participants (distance in seconds estimated, respectively, at 1 *SD* above and below the mean Arab Int:Ind ratio). For the American participants, the pattern flips: the out-group (i.e., Arab-looking) confederates were perceived as farther by the interdependent than the independent subgroups (estimated at 1 *SD* above and below the mean American Int:Ind ratio). Second, this interaction was modified by actual distance to the confederate such that increasing the distance increased the size of the two-factor interaction. As with the first study, this interaction strongly implies the use of different measurement scales (e.g., expected amount of effort) associated with cultural differences.

## Discussion

Contemporary psychology continues to be composed of diverse discourse communities that do not make substantial connection with the discipline as a whole. These diverse communities of psychologists, which have proliferated in rapid succession, increasingly work under different, often conflicting, conceptions of science (Hoshmand and Martin, 1994)… In some cases, psychologists appear to be more interested in contributing to a subdiscipline or specialty than to psychology as a whole (Staats, 1983; Maclntyre, 1985). In this way, fragmentation has been, and continues to be, as much a part of psychology as any of its pragmatic definitional characteristics such as “the study of behavior” or “the study of cognition.” Indeed, there seems to be no evidence that psychology is united by any explicit conception or theoretical framework. (Yanchar and Slife, 1997, p. 236).

What is psychology? Is it a single, coherent scientific discipline awaiting transformation from the current preparadigmatic state into a more mature unified one? Or, is it a heterogeneous federation of subdisciplines that will ultimately fragment into a multitude of smaller, more specialized fields? This is, in essence, the “to be or not to be” question of the field (Henriques, 2004, p. 1207).

*Psychology is what I call a modern disunified science, with a plethora of diverse and unrelated scientific products but with little investment in unifying those products. The resulting disorganization of knowledge leads people such as Toulmin (1972) to consider psychology a “would-be science.” A science in the early stage of disunity does not have the full power of science, and it is not considered to be a full science. That power and that recognition await the beginning of the science's advancement to unification. Psychology has not begun that arduous journey. That will happen inevitably, in my opinion*. (Staats, 2004, p. 273)

These critical citations do not stand alone. They concisely articulate a contentious meta- theoretical controversy that has been reverberating since the latter decades of the past century (Staats, 1983, 1991, 1999; Kimble, 1989; Sternberg and Grigorenko, 2001; Driver-Linn, 2003; Goertzen, 2008). Psychology is perceived by many as a “house divided,” a fragmented collection of sub-disciplines locked into pigeonholes of disparate theoretical paradigms and levels of construct specification, which makes an integrative understanding of behavior difficult. In fact, this apparent lack of common theoretical principles that spans the array of psychological sub-disciplines has led, in some extreme cases, to the reserved use of the label “scientific” in characterizing psychological inquiry (e.g., Koch, 1993).

In light of this last and serious implication, we present here among the first and most explicit empirical attempts to counteract the disunity problem. We developed and experimentally illustrated an approach to unification (Glenberg, 2010): sensorimotor mechanisms can be exploited to traverse the cognitive, social, and cultural domains of behavior while sidestepping the incommensurable theoretical metaphors dominant in each of these territories. Consistent with this approach, the two studies reported here strongly point to the involvement of the motor system even in one of the most abstractly-framed areas of human behavior: culture.

By bringing together findings from the cultural and motor-simulation literatures, we predicted that people with interdependent self construals would anticipate needing less motor effort to interact with in-groups than with out-groups. In contrast, people with independent self construals would anticipate more similar motor effort to interact with in-group and out-group members. We took advantage of the visual signature of motor effort (Proffitt, 2006) to examine this cultural motor-effort hypothesis. Based on Proffitt's work, we expected inflated reports of visual distance to be associated with greater expected effort.

Study 1 confirmed the prediction using two different means of distance estimation, estimated time to walk to a target and estimated number of sticks to the target. Relative to American independents, interdependent Americans reported a shorter expected time to walk to, and fewer sticks to touch, the American in-group confederates. Study 2 replicated and extended the effects by demonstrating that the interdependent Arab participants perceived their in-group Arab confederates as closer than did the independent Arabs, whereas the same Arab confederates were perceived as farther by the interdependent than by the independent American participants. In both studies, the difference between the estimates of the interdependents and independents grew with actual distance, lending further support to the psychological reality of the proposed cultural motor-effort construct.

These result sets are consistent with our prediction that several of the basic characteristics of the motor system (i.e., it scaffolds action recognition and intention-grasping through simulation; it functions predictively by projecting its future states; and it is sensitive to the cost of looming interactions) extend from the basic cognitive (i.e., visual distance perception, Proffitt, 2006), to the interpersonal (i.e., social support, Schnall et al., 2008), and into the domain of self-construal and inter-cultural contact. Importantly, one and the same bodily mechanism can explain these otherwise diverse human behaviors.

Our findings are not the only demonstration of the principle of embodied psychological unity we are trying to promote. In retrospect, many of the embodied-cognition findings may indirectly support the unifying potential of the bodily mechanisms. For example, the neural circuits responsible for the perception of somatic, visceral pain are (a) implicated in one's own experience of social emotions of seclusion (Eisenberger and Lieberman, 2004), (b) resonate with the perceived pain of others (Immordino-Yang et al., 2009), and (c) this resonance is moderated by personality and cultural factors (Avenanti et al., 2010). As another example, the primary somatosensory cortex (that had long been considered to have a purely epistemic function) was recently found to (a) resonate vicariously with the perceived touch of others (Bolognini et al., 2011), (b) show moderated activity based on the assumed gender of who applies the touch (Gazzola et al., 2012), and (c) shows higher resonance levels when the observed touch is at a cultural in-group's body (Xu et al., 2009). And third, circuits that represent comparative magnitude, intensity, and extent (i.e., spatial-cognitive functions; Dehaene et al., 2003) were found to serve the homologous social function of status and rank recognition and discrimination (Chiao et al., 2009). Yamakawa et al. (2009), using fMRI, showed that a common neural substrate located in the parietal lobe is implicated when participants judge the proximity of objects in the physical space as well as when they judged relationships of kinship of family members and closeness of friends.

The above results may, in fact, take the argument for embodied psychological unity (as exemplified in the current research) to a neurophysiological level. Rather than being a mere metatheoretical necessity, the contention that bodily mechanisms can serve multiple cognitive, social, and cultural functions may be reflective of a foundational principle for the functional and structural organization of the brain. Anderson (2010) presents extensive evidence that over both the phylogenic and ontogenic brain lifetimes, “neural reuse” is commonplace. That is, the same neural structures are re-used for progressively more advanced functions. Thus, much as we have argued that sensorimotor systems may underlie individual, social, and cultural behaviors, neural reuse may be a neurophysiological mechanism for how the brain responds efficiently to the cognitive, social, and cultural adaptive demands. In this way, neural re-use may underlie the re-use of sensorimotor mechanisms that we have demonstrated (see also Immordino-Yang et al., 2010).

There is also a body of literature in social psychology that is consistent with our findings. As one example, van Baaren et al. (2003) examined how interdependence and independence affects mimicry. Consistent with our notions of tuning and motor resonance, they report that interdependents produced more non-conscious mimicry. Although less strongly tied to the mechanisms we propose, there is also evidence that mimicry (produced by motor resonance, we suppose) also extends to positive social interactions beyond the dyad (Ashton-James et al., 2007).

Nonetheless, we acknowledge that much more research is needed to further validate the empirical unification approach proposed here. As noted by a reviewer of a previous version of this article, future research should employ designs that allow for a fully crossed cross-cultural investigation. Adding an American confederate to Study 2, for example, would permit examining the proposed cultural motor-effort hypothesis at the (national/cultural) group level, in addition to the cultural individual-difference level (i.e., self construals of interdependence and independence) examined here. Alternative interpretations should also be ruled out. For example, future studies should directly record the height and walking speed (toward culturally neutral, inanimate targets) of interdependents and independents to eliminate these two potential systematic confounds that could yield results similar to the ones reported here (although the reversal of the effect for Americans across the studies make this alternative unlikely). Furthermore, we need to develop a more explicit, mechanistic account of exactly how an anticipated increase in interaction could be used to scale distance. In much of Proffitt's previous work, the connection is close and specific. For example, throwing a heavy ball increases perceived distance to a target when intending to throw, but not when intending to walk. However, in our research and in Schnall et al. (2008) social and cultural factors that are not specifically related to the effectors affect distance perception. Instead, social factors seems to have a generalized effect.

In conclusion, these results are consistent with the cultural motor-effort hypothesis, albeit with the limitations noted above and the possibility of alternative predictions related to self-construal and prejudice noted in the introduction. The results also suggest that the conceptual tools of embodied cognition can be used to help unify psychology by applying the same mechanistic account for behavior at the level of the individual, the social dyad, and the cultural group.

### Conflict of interest statement

The authors declare that the research was conducted in the absence of any commercial or financial relationships that could be construed as a potential conflict of interest.

## Footnotes

^1^The three blocks corresponded to three types of barriers behind which the participants stood: a physical barrier (a fence), a symbolic barrier (a caution tape), and no barrier. This independent variable was included to test another hypothesis. Because the effect of barrier was not significant and did not interact with other variables, it will not be discussed further.

^2^We had hoped to include a larger sample of Arab participants. Unfortunately given current political realities, many Arab students were not willing to participate in psychological research.
